# Applicability of Vacuum Impregnation to Modify Physico-Chemical, Sensory and Nutritive Characteristics of Plant Origin Products—A Review

**DOI:** 10.3390/ijms150916577

**Published:** 2014-09-19

**Authors:** Elżbieta Radziejewska-Kubzdela, Róża Biegańska-Marecik, Marcin Kidoń

**Affiliations:** Institute of Food Technology of Plant Origin, Poznan University of Life Sciences, Wojska Polskiego 31, Poznan 60-624, Poland; E-Mails: elarad@up.poznan.pl (E.R.-K.); kidon@up.poznan.pl (M.K.)

**Keywords:** texture, nutrients, mass transfer, pro-health compounds

## Abstract

Vacuum impregnation is a non-destructive method of introducing a solution with a specific composition to the porous matrices of fruit and vegetables. Mass transfer in this process is a result of mechanically induced differences in pressure. Vacuum impregnation makes it possible to fill large volumes of intercellular spaces in tissues of fruit and vegetables, thus modifying physico-chemical properties and sensory attributes of products. This method may be used, e.g., to reduce pH and water activity of the product, change its thermal properties, improve texture, color, taste and aroma. Additionally, bioactive compounds may be introduced together with impregnating solutions, thus improving health-promoting properties of the product or facilitating production of functional food.

## 1. Introduction

At present methods are being searched to provide food with high health-promoting value, at the same time characterized by desirable sensory attributes. Vacuum impregnation (VI) is a recently developed food processing technique based on diffusion. VI is often confused with osmotic dehydration, although these techniques are completely different. In the case of vacuum impregnation, the mass transfer occurs as a result of mechanically induced difference in pressures, while osmotic dehydration is based on the difference in concentrations between the intracellular liquid in the food material and the solution. Osmotic dehydration requires the use of hypertonic solutions and the process takes place at atmospheric pressure. The main effect is partial water removal from the material, which moves in direction of the concentration gradient. This technique is considered to posses several disadvantages, such as long duration of process (up to a few hours), penetration of the solution inside the material or possible leaching of nutrients from tissues. Additionally, sugar or salt—The most common compounds of osmotic solutions—Change the taste significantly and decrease the nutritional value of food. However, these issues do not occur in the case of vacuum impregnation. In this method the injection of the external solution into the material is the main goal. To achieve this, the application of vacuum is the only necessity, hence the solution may be isotonic. The process is notably faster compared to osmotic dehydration (a few or several minutes). During VI, the solution containing different compounds penetrates into the matrix of food tissue. This may significantly change or improve food properties. The compounds are introduced mainly into intracellular spaces and capillaries of the material in order to provide various advantages, such as an increased nutritive value (e.g., enrichment with polyphenols, probiotics or micronutrients), extension of shelf life (e.g., reduction of pH) or modification of sensory attributes (e.g., introduction of sugar) [1,2,3,4]. Impregnation is an operation frequently applied as a stage in pretreatment, e.g., in minimal processing, drying, freezing or production of fruit and vegetable preserves. Thus, it is essential to determine the potential of this technique to modify physico–chemical characteristics and sensory attributes of products, as well as to select appropriate parameters to ensure the desired effect [5].

## 2. The Course of the Vacuum Impregnation Process

**Figure 1:**
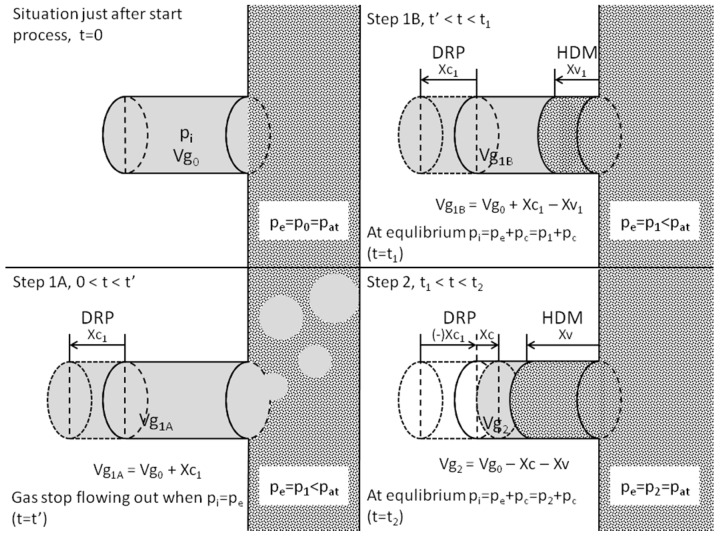
Hydrodynamic mechanism (HDM) and deformation-relaxation phenomena (DRP) contribute to the filling of ideal capillary with liquid during vacuum impregnation [6]. (*t*—Time; *t*’—Time required for internal and external pressure become equal; *t*_1_—Time of vacuum applied (vacuum time); *t*_2_—Time of atmospheric pressure (relaxation time); *p*_0_—Initial pressure; *p*_1_—Vacuum pressure; *p*_2_—Final pressure; *p*_i_—Internal pressure; *p*_e_—External pressure; *p*_c_—Capillary pressure; *p*_at_—Atmospheric pressure; *V*_g0_—Initial volume of gas trapped into the capillary; *V*_g1A_, *V*_g1B_, *V*_g2_—Volume of gas trapped into the capillary after each step of vacuum impregnation; *X*_c1_—Increment of volume of gas trapped into the capillary as result of DRP; *X*_c_—Decrement of volume of gas trapped into the capillary as result of DRP; *X*_v1_—Partial decrement of volume of gas trapped into the capillary as result of HDM; *X*_v_—Decrement of volume of gas trapped into the capillary as result of HDM).

During typical vacuum impregnation the free spaces and capillaries of the material are filled due to a mechanically induced difference in pressure. The process consists of two stages: The phase of reduced pressure and the phase of atmospheric pressure. Impregnation of the material occurs as a consequence of two phenomena: hydrodynamic mechanism (HDM) and deformation–relaxation phenomena (DRP), which lead to the filling of intracellular capillaries (Figure 1).

After immersion of the material in solution (*t*_0_), the pressure inside (*p*_i_) and outside (*p*_e_) the capillary are equal to atmospheric pressure (*p*_i_ = *p*_e_ = *p*_at_) and they equalize. The initial volume of the capillary (*V*g_0_) is filled with gas (Figure 1, Step 0). In the first phase of the process the pressure is reduced (*p*_1_ < *p*_at_). As a result of the difference in pressures, the gas is removed from the capillary. Reduced pressure acting from the outside causes the deformation and expansion of the capillary, which is the first part of the deformation–relaxation phenomenon (DRP). The volume of the capillary is increased (*V*g_1A_ = *V*g_0_ + *X*c_1_). This stage lasts until pressure equilibrium (*p*_i_ = *p*_e_) is reached (Figure 1, Step 1A). Next, the capillary starts to be partially filled with liquid, as a result of the HDM. The pressure inside the capillary increases slightly, while the free volume inside it decreased to the value *V*g_1B_ = *V*g_0_ + *X*c_1_ − *X*v_1_ (Figure 1, Step 1B). In the second phase of vacuum impregnation the pressure returns to the atmospheric value. This causes the transition of the DRP to the relaxation phase. The capillary shrinks to an even greater extent than before the start of the process. At the same time, as a result of the action of capillary pressure and decompression, an intensive inflow of liquid from the outside to the inside of the capillary is observed and the final volume of gas inside it decreases to *V*g_2_ = *V*g_0_ − *X*c − *X*v (Figure 1, Step 2). The relaxation phase is particularly important from the practical point of view, since tissue impregnation occurs at this stage. Removal of vacuum should not be too rapid, since the excessively fast pressure equalization may lead to closure of the capillary vessels and inhibition of the hydrodynamic mechanism [6,7].

The course of the vacuum impregnation process is affected by numerous factors, both internal and external. Internal factors include properties of the impregnated tissue, such as its structure (porosity), mechanical properties, the shape and size of the capillary as well as the shape and the size of the sample. The most important external factors influencing the rate of impregnation include the level of applied vacuum pressure, the composition and concentration of the impregnating solution, temperature, the of the solution amount to product ratio, mixing, the time required to reach vacuum and the duration of vacuum treatment as well as the restoration of atmospheric pressure [1,5,6].

## 3. The Application of Vacuum Impregnation to Modify Physico–Chemical Properties and Sensory Attributes of the Product

Due to the high volume of intracellular spaces in tissues of fruit and vegetables, the following agents may be introduced to its interior: cryoprotectants, inhibitors of enzymatic browning, enzymes, compounds enhancing tissue compactness and inhibiting its softening or compounds reducing water activity [8,9,10,11,12,13] (Table 1). Thus, the use of vacuum impregnation may contribute to a new product quality, connected both with physico–chemical properties and sensory attributes.

**Table 1:** Examples of applications of vacuum impregnation to modify physico–chemical properties and sensory attributes of products.

Raw Material	Composition of Vacuum Impregnation Solutions	Process Parameters	Effect	References
peppers (slices of 15 cm in length and 1 cm in width)	lactic acid solution (pH 2.70)	solution:sample mass ratio of 5:1;	increase of the acidification degree in peppers	[10]
		*p*_1_ 20 or 40 kPa		
		*t*_1_ 2 or 5 min		
		*t*_2_ 10, 15 and 30 min		
mushrooms (*Agaricus bisporus*) (cut in half)	lactic acid solution (pH 3.05)	solution:product ratio of 8:1;	increase of the acidification degree in mushrooms	[14]
		*p*_1_ 20 and 40 kPa		
		*t*_1_ 2 min		
		*t*_2_ 20, 40, 60, 120, 240, 300, 360 and 720 min		
zucchini (slices of 1.5 cm in thickness and a diameter of 2.0 cm; the average weight of each slice was 5 g)	lactic acid solution (pH 2.70)	solution:product mass ratio of 8:1;	increase of the acidification degree in zucchini slices	[15]
		*p*_1_ 20 and 40 kPa		
		*t*_1_ 2 min		
		*t*_2_ 20, 40, 60, 120, 240, 300, 360 and 720 min		
papayas (cut into 4 × 2.5 × 0.5 cm pieces (length × width × thickness))	55% and 65% (*w*/*w*) sucrose solution	*p*_1_ 5 kPa	decrease of *a*_w_	[16]
		*t*_1_ 10 min at 30 °C		
strawberry	65% (*w*/*w*) sucrose solution	steam blanching or microwave and osmotic dehydration at atmospheric pressure or pulsed vacuum treatments	decrease of *a*_w_	[17]
		*p*_1_ 5 kPa		
		*t*_1_ 5 min at 30 °C		
rabbiteye blueberries	aqueous sucrose solutions (600 g/kg)	solution:product ratio of 1:1;	shortenning of dehydratation time in comparison with soaking at atmospheric pressure	[18]
		*p*_1_ 88 kPa		
plum (cut in slices of 4 × 1 × 1 cm, weighting approximately 10 g)	40°, 50° and 60° Brix sucrose solution	solution:product ratio of 10:1;	new product with good visual quality and satisfactory shrinkage	[19]
		*t*_1_ 10 min		
apples cv. (cultivar). Granny Smith (cylindrical samples (2 cm height and diameter))	rectified grape must (hypertonic solutions: 65°, 50° and 30° Brix) and 3% (*w*/*w*) high methoxyl pectin solutions	*p*_1_ 5 kPa	improvement of mechanical and structural properties of tissue, notable reduction of freezable water which could improve fruit resistance to freezing damage	[13]
		*t*_1_ 5 min		
		*t*_2_ 25 min in higher solution viscosity		
		*t*_2_ 55 min		
strawberry (10 mm slices)	50% (*w*/*w*) high fructose corn syrup or 3% (*w*/*w*) high methoxyl pectin solution containing calcium and/or zinc	*p*_1_ 7 kPa	improvement of textural quality and reduced drip loss of frozen-thawed strawberries	[20]
		*t*_1_ 15 min		
		*t*_2_ 30 min		
spinach (rectangular 3.0 cm long, 0.5 cm wide and 0.06 cm thick)	40% (*w*/*w*) trehalose solution	pulsed electric fields (580 V/cm) in combination with vacuum impregnation	improvement of freezing tolerance of spinach leaves	[21]
		*p*_1_ 86 kPa		
		*t*_1_ 20 min		
		*t*_2_ 150 min		
apple samples cv. Granny Smith (cylindrical samples (8 cm height and 2 cm diameter))	sucrose isotonic solution	*p*_1_ 50 kPa	increase of thermal conductivity	[22]
		*t*_1_ 10 min		
		*t*_2_ 20 min		
zucchini (slices 0.5-cm thick)	maltodextrine solution (7.5%–9%, 10%), NaCl (0%–5%) and CaCl_2_ (0–1000 mM)	product:solution ratio of 1:3.3;	improvement of solute and water gain and limitation of textural and microstructural changes	[23]
		*p*_1_ 2.5 kPa		
		*t*_1_ 10 min		
		*t*_2_ 30 min		
eggplant, carrot, oyster mushroom	33 g sucrose and 20 g calcium lactate solution in isotonic solution	*p*_1_ 5 kPa	notable impact on mechanical behaviour of eggplant and carrot, no effects in oyster mushroom	[1]
		*t*_1_ 10 min		
		*t*_2_ 10 min		
apple samples cv. Granny Smith	CaCl_2_ solution (0.6%, 2.0% or 4.0% (*w*/*w*))	*p*_1_ 9.3 and 59.9 kPa	improvement of texture	[24]
		*t*_1_ 4 min		
		*t*_2_ 5 min		
apple cv. Jonagold (cut into 1 cm thick slices)	10 mg/L ascorbic acid, 0.05 mg/L 4-hexylresorcinol, 5 mg/L calcium chloride and 200 mg/L sucrose	*p*_1_ 8 kPa	the same effect of dipping and vacuum impregnation regarding hardness	[25]
		*t*_1_ 5 min		
		*t*_2_ 5 min		
apples cv. Granny Smith (1 cm cubes) strawberries (cut in halves) and raspberries	high methylated pectin solution preparation up to 3% (*w*/*w*) and/or CaCl_2_, up to 6.5% (*w*/*w*)	*p*_1_ 6.6 kPa	limitation of loss in fruit firmness following pasteurization	[26]
		*t*_1_ 2 min		
strawberries (cv. Elsanta and Darselect) (cut in halves)	high methylated pectin (from *Aspergillus aceleatus*) containing 100 U/mL, 0.5% (*w*/*w*) CaCl_2_·2H_2_O 1% and 3% (*w*/*w*) of apple pectin	*p*_1_ 1 kPa	limitation of structural damage during subsequent rapid freezing processes	[27]
		*t*_1_ 5 min		
peaches (cut in halves)	pectin methylesterase together with CaCl_2_ (100 mg/L)	*p*_1_ 85 kPa *t*_1_ 30, 60, 90, 120 min	increase of firmness in canned peaches	[28]
eggplants (slices of 1 cm trick)	pectinmethyl-esterase derived from *Aspergillus niger* and extracted from orange and grapefruit and 4000 ppm CaCl_2_·2H_2_O	1st method:	increase of firmness in impregnated eggplants	[29]
		*p*_1_ 68 kPa		
		*t*_1_ 15 min at 30 °C		
		2nd method: pulsed vacuum impregnation		
		*p*_1_ 85 kPa		
		*t*_1_ 5 min		
		release vacuum to atmospheric pressure for 1 min reapply vacuum for 5 min and release for 5 min		
watercress (leaves were selected diameter 1.4 cm)	winter flounder antifreeze protein type I solution (1 mg/100 mL AFP-I ultra pure water)	*p*_1_ 51, 58, 68, 85 and 101 kPa	smaller ice crystals in AFP-I impregnated (58 kPa, for 5 min) frozen samples	[30]
		*t*_1_ 5 min		
strawberry	12 g/100 g trehalose solution; 0.2 g/100 g solution unpasteurized cold acclimated winter wheat grass extract as a source of AFP and 12 g/100 g trehalose and 0.2 g/100 g unpasteurized cold acclimated winter wheat grass extract	*p*_1_ 86 kPa	improvement of freezing tolerance of strawberry	[31]
		*t*_1_ 5 min		
strawberry slices	CaCl_2_ solution (1, 10, 100 mM); spermine solution (1, 10, 100 mM); spermidine (1, 10, 100 mM); putrescine (1, 10, 100 mM);	*p*_1_ 16.9 kPa	effect of spermine and spermidine on the increase of firmness, whereas putrescine was not as effective	[32]
		*t*_1_ 8 min		
carrots (cv. Nantesa) slices (20-mm diameter, 10 mm thick)	chitosan (1%, *w*/*v*) dispersed in aqueous solution of glacial acetic acid (1%, *w*/*v*), at 40 °C	*p*_1_ 5 kPa	improvement of sample resistance to water vapor transmission, better preservation of color and mechanical response during cold storage	[33]
		*t*_1_ 4 min		
		*t*_2_ 2 min		
pineapple (slices 1 cm thickness)	chitosan- or casinate-based film-forming emulsions	ratio of the weight of coating solution:sample: 20:1;	extension of shelf-life in pineapple-cereal system for caseinate based coating	[34]
		*p*_1_ 5 kPa		
		*t*_1_ 3 min		
		*t*_2_ 2 min		
pear (*Pirus communis* cv. *Blanquilla*) (cylinders 2 cm height, 2 cm diameter)	isotonic sucrose solution (14° Brix) containing trisodium citrate 2-hydrate, sodium l-ascorbate, ethylenediamine tetraacetic acid 2-hydrate disodium salt and calcium lactate 5-hydrate and 4-hexylresorcinol	solution: fruit ratio of 20:1;	ascorbate and calcium lactate in impregnated solution were the most effective for extending the shelf life of pear	[35]
		*p*_1_ 5 kPa		
		*t*_1_ 5 min		
		*t*_2_ 10 min		
apple cv. Jonagold (1-cm thick slices)	ascorbic acid, citric acid, 4-hexylresorcinol, sodium chloride, calcium chloride, sodium lactate, calcium lactate and sucrose solutions	*p*_1_ 7 kPa	effective inhibition of browning and softening of apple slices during storage by 1% ascorbic acid, 0.005% 4-hexylresorcinol, 0.5% calcium chloride, 20% sucrose in impregnated solution	[36]
		*t*_1_ 5 min		
		*t*_2_ 5 min		
button mushrooms (slice thickness was 6.5 mm with a 3 to 5 mm cap length)	2 g/100 g ascorbic acid + 1 g/100 g calcium lactate solution; 2 g/100 g citric acid + 1 g/100 g calcium lactate; 1 g/100 g chitosan + 1 g/100 g calcium lactate solution; and 1 g/100 g calcium lactate solution	*p*_1_ 6.7, 10.0, 13.3, 16.7 kPa	vacuum impregnation with ascorbic acid and calcium lactate at 6.7 kPa for 5 min and atmospheric restoration time of 5 min was the most effective to limit adverse changes of color in sliced button mushrooms	[37]
		*t*_1_ 5 and 10 min		
		*t*_2_ 5 and 10 min		
litchi cv. Rose	502 g/kg sucrose solution containing 4.9 g/kg cysteine + 20 g/kg ascorbic acid + 0.134 g/kg 4-hexyl resorcinol and 502 g/kg sucrose solutions also contained 20 g/kg calcium lactate and 1 g/kg potassium sorbate	*p*_1_ 76 kPa	samples were sensory acceptable up to 24 days	[38]
		*t*_1_ 10 min		
		*t*_2_ 10 min		
apple sticks	mass ratio of fruit:syrup was 1:17; fructose isotonic solution (14.0°–15° Brix) containing ascorbic acid (0.5% *w*_t_/*w*_t_) and dry, food-grade green apple flavoring (0.5% *w*_t_/*w*_t_)	*p*_1_ 28 kPa	aroma enrichment	[39]
		*t*_1_ 5 min		
		*t*_2_ 2.5, 5, 12.5 min		
olive fruits cv. Domat	NaCl (3%), NaOH (1.5%) and NaOH (1.5%) + NaCl (3%) solutions	*p*_1_ 68 kPa	shortening the duration of debittering process	[40]
apples cv. Granny Smith and Stark Delicious	higher values of hardness, crispness, juiciness and sourness in vacuum impregnated Granny Smith apples	the solution:fruit ratio was 11:1;	higher values of hardness, crispness, juiciness and sourness in vacuum impregnated Granny Smith apples	[41]
		*p*_1_ 10 kPa		
		*t*_1_ 30 min		
		*t*_2_ 5 min		

*p*_1_—vacuum pressure in the Vacuum impregnation (VI) process; *t*_1_—time in reduced pressure; *t*_2_—time in atmospheric pressure.

### 3.1. Reduction of pH

A major factor influencing microbiological safety of products is connected with pH. Lowering of pH value reduces thermal resistance of microorganisms and their growth rate, while it also prevents out-growth of spores. Introduction of organic acids to plant tissue by vacuum impregnation facilitates the reduction of pH in the raw material. At a lower pH value, the microorganisms use more energy to maintain neutral pH within their cells and use less energy for growth [42]. Lowering the pH value also leads to reduced thermal resistance of microorganisms. At present, the raw material is acidified by blanching in water or soaking, using the difference in the concentration of hydrogen ions between the solution and raw material as the driving force of this process. It is frequently a long-term process, particularly in the case of soaking. As a result of vacuum impregnation, the tissue porosity is enhanced through expansion of gas trapped in pores; thus, a greater volume of raw material is available in the impregnation process during the restoration of atmospheric pressure. In the study reported by Derossi *et al.* (2010) it was observed that the reduction of pH value was greater during vacuum impregnation of pepper slices than in the case of blanching at atmospheric pressure [10]. The authors explain this phenomenon by an increase in the diffusion rate of hydrogen ions as a result of an increase in the contact surface between lactic acid solution and raw material tissue. This indicates a direct correlation between a decrease in pH and the duration of vacuum pressure and relaxation time. By increasing the time of the vacuum impregnation from 2 to 5 min (in particular at a pressure of 20 kPa) and relaxation time from 10 to 30 min, Derossi *et al.* (2010) obtained a significant decrease in pH of the tissue material [10]. The effect of impregnation time on the amount of solutes impregnating an apple, mushroom, banana, strawberry and apricot was also researched by Fito *et al.* (1996) [6,43]. The authors used a vacuum time of 5–20 min and relaxation time of 5–15 min. Neither of the abovementioned time periods was found to have a significant effect on effective porosity values, however various effects on the volumetric fraction of the sample occupied by the liquid were observed depending on the raw material [6]. It can be assumed that the tissue structure plays a very important role, not only due to the total porosity, but also regarding the size and shape distribution of pores as well as their communications between themselves and with the external liquid. Salvatori *et al.* (1998), studying the effects of VI on pore volume impregnated with an external (osmotic) solution and on the deformation phenomena of several fruits, found that strawberries exhibited a negligible impregnation level, even though this type of fruit showed greater porosity fraction (6.3%) in comparison with kiwi fruit and peach [44]. These differences can be attributed to the microscopic properties of the strawberry tissues, such as high tortuosity of the internal pathways and/or size and shape of pores which hindered the influx of the external solution. This may be indicated by the results obtained by Mújica-Paz *et al.* (2003), which are contradictory to those reported by Fito *et al.* (1996) [6,45]. In the framework of their study, Mújica-Paz *et al.* (2003) established that the effect of vacuum time (3–45 min) on the volume of isotonic solution impregnating slices of mango, apple, papaya, banana, peach and melon depended significantly on the vacuum time, except for apple [45].

The degree of tissue impregnation also depends to a great extent on the level of applied reduced pressure [46]. Mújica-Paz *et al.* (2003) stated that the volume of tissue impregnated with the solution increased with the increase in vacuum pressure for apple, papaya and melon [45]. However in the case of zucchini slices, the value of the applied pressure of 20 or 40 kPa had no significant effect on tissue acidification [15]. In turn, the degree of tissue impregnation for banana, peach, mamey and mango was decreased at higher pressure values [47]. Fito *et al.* (1996) explained this by irreversible tissue deformation, and thus reduction of free space for the solution. It may be assumed, that the degree of tissue impregnation is associated to a considerable extent with porosity, the size and shape of the pores as well as mechanical properties [6]. A considerable tissue rigidity may be reduced by the deformation-relaxation phenomenon. This is indicated by a significant effect of raw material structure on the vacuum impregnation process and thus the modified quality of the product. The effect of vacuum and relaxation times on the process of tissue impregnation by the solution is not entirely clear. The conducted studies give contradictory results.

### 3.2. Reduction of Water Activity (a_w_)

Vacuum impregnation may also be used to reduce water activity in tissue of fruit and vegetables. This technique may be applied e.g., as the first stage of osmotic dehydration. The application of vacuum during osmotic dehydration (vacuum osmotic dehydration—VOD) improves capillary flow and mass exchange. It is most probably a result of surface tension at the solid–solution interface [46,47,48]. The application of vacuum impregnation for a short time (5–15 min), followed by long-term osmotic dehydration, is referred to as pulsed vacuum osmotic dehydration (PVOD). As a result of this process, the intracellular spaces are filled with osmotic solution initiating osmotic dehydration. An increase of the solid content in tissue is inversely proportional to the concentration of osmotic solution [48]. The raw material is impregnated by the application of solutions with a lower viscosity, whereas high viscosity hinders penetration of the solution into tissues, resulting in water loss. Mújica-Paz *et al.* (2003) in their researches found that the lowest final *a*_w_ levels in apple and mango can be achieved with 50° Brix syrup and vacuum pressure of 67.4 kPa and in melon with 57° Brix and 59.3 kPa [47]. The difference in the preservation of fruit and vegetables during the dehydration process at a reduced pressure also influences the porosity of the raw material [47]. Mújica-Paz *et al.* (2003) studied the effect of vacuum pressure (13.5–67.4 kPa) on the weight reduction (WR),water loss (WL) and solid gain (SG) of apple, melon and mango slices used a hypertonic solution for a *t*_1_ = *t*_2_ = 10 min [47]. For melon and mango samples showed a positive WR, stating that fruits lost a significant fraction of their weight; instead, apples showed a negative WR values for solution concentration lower than 50° Brix, stating a weight gain. The negative values observed for apple samples were explained on the basis of their high porosity fraction (~27.3%). In these conditions, the high free volume of apples increased the impregnation level more than water loss leading to an increase of weight of samples [47]. However, for a solution concentrate 50° Brix, the pressure gradient promoted the outflow of water from the apple microstructure. Similar effect of the osmotic solution concentration was observed from other authors [13,49].

Vacuum osmotic dehydration may be applied, e.g., to improve quality of dried, frozen or minimally processed products. During the study on vacuum impregnation of papaya using a hypertonic sucrose solution (55° or 65° Brix), Moreno *et al.* (2004) observed the most effective reduction of water activity when a solution with a higher extract content was applied [16]. Moreover, the authors recorded an improvement in texture and taste of papaya in comparison to those of the raw material, which was dehydrated at atmospheric pressure. Moreno *et al.* (2000) also investigated the effect of dehydration in vacuum or at atmospheric pressure, carried out after steam or microwave blanching, on quality of minimally processed strawberries [17]. The authors used a hypertonic sucrose solution (65° Brix) in osmotic dehydration. Pulsed osmotic dehydration of strawberries following steam blanching was the most effective method to reduce *a*_w_ due to the highest sucrose gain during osmotic treatment. However, this resulted in a deterioration of texture and color of raw material, at the simultaneous maintenance of the greatest microbiological stability of the sample.

In the case of drying, vacuum osmotic dehydration in a hypertonic solution may result in a reduction of raw material moisture content and a shortening of drying time, thus contributing to an improvement of dried material quality. Pallas *et al.* (2013) reported a significant reduction of drying time as a result of vacuum osmotic dehydration of rabbiteye blueberries in a sucrose solution (60° Brix) [18]. Application of vacuum osmotic dehydration for 70 min resulted in a reduced moisture content of raw material to the same level as after 24 h of dehydration at atmospheric pressure. In turn, pulsed vacuum osmotic dehydration of prunes (which were blanched with KCl) using a hypertonic solution provided a product with a reduced final moisture content and good visual quality, as well as satisfactory drying shrinkage [19].

In the case of freezing, a partial removal of water may reduce the content of frozen water and provide a more stable product as a result of an increase in glass transition temperature at maximum cryoconcentration of the liquid phase of the product [13]. The phase transition of water in the raw material frequently results in disrupted tissue integrity and leads to undesirable physico-chemical changes. Pretreatment by vacuum impregnation prior to freezing may contribute to a limitation of thawing drip and improvement of texture in frozen fruit and vegetables. When a hypertonic solution is used during impregnation, the raw material is osmotically dehydrated, resulting in cryostabilisation due to a reduction of water content. Xie and Zhao (2004) used a high-fructose corn syrup (50%) or high-methylated pectin (3%) to impregnate strawberries prior to the freezing process [20]. They found that vacuum impregnation resulted in a strengthened structure and reduced water drip in thawed strawberries. High-fructose corn syrup decreased the amount of frozen water in tissue, while high-methylated pectin penetrated into intracellular spaces protecting fruit against freezing damage. Martínez-Monzó *et al.* (1998) used a concentrate of grapefruit must and pectin solution to impregnate apples [13]. The authors observed reduction of water content in the raw material, which may result in an increased tissue resistance to freezing damage. In the case of pectin addition to the solution, they suggested a potential hardening of the cell structure due to the formation of a polysaccharide gel. Phoon *et al.* (2008) stated that vacuum impregnation with a 40% (*w*/*w*) trehalose solution combined with pulsed electric field treatment by a CythorLab™ electroporator (Aditus AB, Lund, Sweden) facilitates improved the freezing tolerance of spinach [21]. Trehalose, apart from decreasing the chemical potential of water and the freezing point in the cytosol, is well-suited for stabilizing the cell membrane through hydrogen bonding between the hydroxyl groups on the sugar and the polar residues in phospholipids due to the hydrophilic nature of sugars. This prevents dehydration effects in membranes [50]. The combined unit operations probably allowed trehalose to be present in both the extracellular and intercellular spaces. Trehalose would then be able to protect the cell from both sides.

The abovementioned references indicate that in order to reduce the water activity using vacuum impregnation, solutions with extract content ranging from 30° to 65° Brix should be used. The best effect can be achieved with a higher content of the extract but the relaxation time should also be prolonged because of the high viscosity of the solution. The applied vacuum pressure was 5 kPa for minimally processed products and ranged from 5 to 88 kPa for dehydrated food, at the vacuum time of 5 to 20 min. The relaxation time for minimally processed products ranged from 10 to 30 min, and for the dehydrated food from a few minutes to a few hours. Sucrose solutions were mainly used during the pre-treatment before drying, while solutions containing cryoprotectants (hypertonic sugar solution) and cryostabilizers (metoxyl high pectin) were employed in the case of freezing.

### 3.3. Changes in Thermal Properties

Vacuum impregnation also seems to be a suitable technique for modifying the thermal properties of fruit and vegetables. Thermal properties of raw material play a significant role during blanching or preservation of the product by pasteurization or sterilization. The conductivity and thermal diffusion coefficient are determined to a considerable extent by the composition and structure of the product. Modification of its structure by vacuum impregnation applied prior to thermal treatment may improve the efficiency of heat conduction and enhance the quality of the product. Martínez-Monzó *et al.* (2000) in the case of vacuum impregnation with an isotonic sucrose solution recorded an increase in thermal conductivity of apples by 15%–24%, while changes in the diffusion coefficient were slight [22]. Fito *et al.* (2000) explained that an increase in thermal conductivity may be a result of the replacement of gas in intracellular spaces with the solution, while a slight increase in the diffusion coefficient may be caused by a simultaneous increase in density [51]. Shortening the duration of thermal treatment during the technological process as a result of the substitution of gas with a liquid in the intracellular space may notably contribute to an improvement in the quality of both products, *i.e.*, dried or frozen material, and fruit and vegetable preserves.

### 3.4. Improvement in the Structure of Fruit and Vegetables

Vacuum impregnation may also be used in fruit and vegetable processing to modify the structure of raw material. Most processes such as blanching, pasteurization, sterilization and freezing cause irreversible damage to plant tissue. A frequently described method to enhance the texture and limit cell liquid drip is to apply the pretreatment of raw material, focused on the introduction of calcium ions inside the tissue [52,53]. The mechanism of this process is explained by binding (chelation) of calcium ions by carboxyl groups of pectin, which leads to gel formation at low pH [54]. It seems that vacuum impregnation may facilitate a more effective impregnation of tissue with calcium ions than blanching or soaking. Strengthening of raw material structure by vacuum impregnation was reported e.g., by Occhino *et al.* (2011) when vacuum impregnation of zucchini with a calcium chloride solution and a mixture of calcium chloride, maltodextrin and sodium chloride was studied [23]. The effect of enhanced rigidity and brittleness of tissue in carrot and eggplant as a result of vacuum impregnation with a sucrose and calcium lactate solution was also observed by Gras *et al.* (2003) [1]. Del Valle *et al.* (1998) made an attempt at the application of vacuum impregnation to improve the texture in minimally processed apples [24]. The authors applied, e.g., pretreatment of raw material by vacuum impregnation with a calcium chloride solution and impregnation preceded by blanching in the high temperature short time. They stated that a decrease of pressure during impregnation from 59.9 to 9.3 kPa caused cell damage. However, when comparing the sample impregnated with water with that impregnated with a calcium chloride solution, they observed improved mechanical properties of the tissue connected with an addition of calcium ions to the solution. Application of vacuum impregnation after blanching did not provide the expected effect of texture improvement in apples, irrespective of the volume of the applied pressure and concentration of calcium ions in the solution (0.2%–4.0% *w*/*w*). This may be connected with the removal of gases and partial filling of the intracellular space by the liquid during thermal treatment. As a result, a lower amount of calcium ions may penetrate to the tissue during vacuum impregnation. Those authors also investigated the texture of apples, which were subjected e.g., to blanching (60 min at 40 °C, 15 min at 55 °C and 15 min at 65 °C) in 0.6% calcium chloride solution or vacuum impregnation with a 2% calcium chloride solution followed by osmotic dehydration. An improved texture of apples in comparison to the control (with no pretreatment) was observed in the sample impregnated and blanched for 15 min at 55 °C. This was connected with a better preservation of tissue microstructure. However, the results of studies concerning the effect of vacuum impregnation with calcium ions on the structure of plant tissue are not conclusive. Attempts to apply calcium chloride in combination with ascorbic acid, 4-hexylresorcinol and sucrose in order to improve the texture in minimally processed apples were also made by Biegańska-Marecik and Czapski (2007) [25]. The authors compared the effect of three pretreatment methods: soaking, blanching and vacuum impregnation, on the mechanical properties of the minimally processed product. The greatest changes of texture in apple tissue were found in samples subjected to blanching. Soaking and vacuum impregnation of samples preserved hardness of tissue comparable to that of fresh raw material (the control). During sample storage the best texture was found for apples subjected to soaking. In turn, Anino *et al.* (2006) used an isotonic solution of glucose with an addition of calcium lactate and calcium gluconate in vacuum impregnation of apples [55]. These calcium salts are frequently used in vacuum impregnation of fruit and vegetables due to their good solubility at room temperature and neutral taste. However, the authors found no effect of tissue impregnation with calcium ions on the improvement of texture. In the tested samples, they recorded numerous examples of cell damage and plasmolysis. However, they observed a greater rigidity of tissue in apples after vacuum impregnation than after the process conducted at atmospheric pressure.

A promising method of countering changes in mechanical properties of tissue in fruit and vegetables seems to be provided by a combination of pectin methylesterase (PME) with calcium ions in the impregnating solution. Vacuum impregnation technology allows for a contact between the enzyme (PME) and the substrate in solid food. Pectin methylesterase catalyzes the hydrolysis of methyl groups esterifying pectin, a major constituent of plant cell walls. Its action releases free carboxylic acids, which may then interact with calcium. This chelation of calcium by the free carboxylic acid moieties of pectin molecule would form a gel strengthening the cell wall structure and thus increasing the firmness of fruit. The recommended procedure for enzyme incorporation is to soak the fruit pieces in an enzyme solution for a time period between a few minutes to 1 h at normal pressure. Guillemin *et al.* (2006) showed that vacuum-impregnation can be used to add exogenous PME to fruit pieces more rapidly and more homogeneously than soaking in order to improve the firmness of thermally treated fruit [56]. The effect of structure strengthening in pasteurized apples, strawberries and raspberries as well as frozen strawberries as a results of vacuum impregnation with a solution containing PME and calcium ions was reported e.g., by Degraeve *et al.* (2003), Suutarinen *et al.* (2000) and Van Buggenhout *et al.* (2006, 2008) [26,27,54,57]. Improvement of the texture was also observed by Javeri *et al.* (1991) during the impregnation of vacuum blanched peach halves, which were pasteurized afterwards. The increase in tissue hardness observed by those researchers was not proportional to the continuous increase in the concentration of PME and calcium ions in the solution [28]. The presence of calcium ions activates PME, an enzyme which causes disesterification of pectin and thus facilitates the binding of calcium ions to free carboxyl groups. The effect of pectin disesterification as a result of vacuum impregnation in apple tissue using a solution containing PME and calcium ions was confirmed by a study of Guillemin *et al.* (2006) [56]. The authors found a reduction of carboxyl groups esterified with methyl alcohol in the tested tissue in the range from 85% to 45% in comparison to water-impregnated apples.

The relevant literature data suggest that in the case of freezing an alternative to calcium ions and pectin methyl esterase may be provided by the application of antifreeze protein type I in the impregnating solutions [30]. Antifreeze proteins may decrease the freezing point of aqueous solutions below the melting point, inhibit ice recrystallization, and suppress or modify ice crystal growth [58]. Application of antifreeze proteins in frozen food may inhibit recrystallization during freezing, storage, transport and thawing, thus preserving food texture by reducing cellular damage, while also minimizing the loss of nutrients by reducing drip [59]. Antifreeze proteins I were used in the impregnation of watercress e.g., by Cruz *et al.* (2009) [30]. Based on the scanning electron microscopy (SEM) (JEOL JSM-5600 LV, Tokyo, Japan) analysis, the authors found better cell wall definition and a rounded cell shape with smaller ice crystals compared to the control impregnated with water. Antifreeze protein from cold-acclimated wheatgrass was also applied by Velickova *et al.* (2009) [31]. During vacuum impregnation of whole strawberries with a solution containing cold-acclimated wheatgrass and trehalose the authors recorded increased tissue hardness and limitation of thawing drip.

The use of vacuum impregnation allows for introduction of other structure-forming compounds, e.g., polyamines, into the plant tissue, while its structure may also be modified due to the formation of edible coats. Polyamines exhibit properties similar to those of calcium ions. They may bind with cell walls and pectins found in the middle lamella [60]. On the other hand, they may inhibit the synthesis of ethylene in damaged tissue and reduce the activity of enzymes responsible for its softening [61]. The use of vacuum impregnation technique to introduce these compounds to the tissue of strawberries was tested by Ponappa *et al.* (1993) [32]. The authors stated that vacuum impregnation with spermine and spermidine caused a significant improvement in the hardness of strawberries stored at a temperature of 1 °C. No such effect was recorded in the case of putrescine. In their studies, the authors observed no interdependence between the level of endogenous polyamines in the tissue and its texture. In turn, Vargas *et al.* (2009) applied vacuum impregnation in order to coat a carrot with chitosan-based edible film [33]. They obtained better adherence of the coating to tissue than in the case of immersion in film-forming dispersions at atmospheric pressure. The produced film was thicker and uniformly distributed over the sample surface. Coating by vacuum impregnation resulted in an improvement of mechanical properties and color of carrot slices during storage. Vacuum impregnation was also used by Talens *et al.* (2012) for the formation of edible coatings [34]. The authors applied this technique to coat partly dehydrated pineapples, which constituted a component of a dry fruit and cereal product. They used caseinate and chitosan to produce the coating. A problem with products composed of multiple components differing in their water activity is associated with migration of water, which may cause physical and chemical changes, thus reducing the shelf life of the product. The primary quality attribute in such products is their crunchiness. Coating of dehydrated pineapples with an emulsion containing caseinate using vacuum impregnation contributed to an extension of shelf life of the product.

### 3.5. Improvement of Color, Aroma and Taste

One of the factors limiting the quality of fruit and vegetable products is their color. Frequently, a change in color is a result of enzymatic browning occurring in plant tissue. The mechanism of this reaction is connected with the activity of enzymes from the group of polyphenol oxidases, which catalyze the oxidation of phenolic compounds with oxygen [62]. Enzymatic browning of plant tissue is most frequently inhibited by blanching or soaking of raw material in a solution of inhibitors. It seems that the method of vacuum impregnation by intensive impregnation of the tissue with a solution of enzymatic browning inhibitors or removal of oxygen from the intracellular space may contribute to the inhibition of adverse changes in product color. A more effective action in the tissue, observed for inhibitors such as citric or ascorbic acids, may constitute an alternative to sulfur compounds (IV); which frequently cause allergic reactions or alter the taste of the product. An example for the application of vacuum impregnation to inhibit enzymatic browning may be provided by a study by Perez-Cabrera *et al.* (2011) [35]. During the impregnation of pears the authors used an isotonic solution containing enzymatic browning inhibitors (ascorbate; 4-hexylresorcinol; EDTA; citrate) with or without an addition of calcium lactate. The most effective limitation of adverse changes in color were observed as a result of vacuum impregnation of pears with a solution containing ascorbate and an addition of lactate. They also recorded an extended shelf life of the minimally processed product to 20 days and inhibition of changes in the mechanical properties of tissue as well as microbial growth. The effect of enzymatic browning inhibition during storage was also observed by Biegańska-Marecik and Czapski (2007) when applying a solution containing ascorbic acid; 4-hexylresorcinol; calcium chloride and sucrose in vacuum impregnation of apple slices [36]. In order to limit adverse changes in color in sliced button mushrooms (*Agaricus bisporus*) during vacuum impregnation Yurttas *et al.* (2014) applied a solution containing ascorbic acid; citric acid; calcium lactate; and chitosan [37]. The best effect was provided by the application of a solution containing ascorbic acid and calcium lactate. In turn, Shah and Nath (2008) observed an inhibition of enzymatic browning both in the case of soaking at atmospheric pressure in a solution containing cystein; ascrobic acid and 4-hexylresorcinol as well as during vacuum impregnation of minimally processed lychee [38]. A similar effect was recorded by Blanda *et al.* (2008) after soaking or vacuum impregnation with a solution containing fructose; calcium chloride dihydrate; ascorbic acid and sodium chloride applied for nectarine halves, which were then subjected to freezing and thawing [63]. It seems that vacuum impregnation is an effective method for inhibiting enzymatic browning. However, sometimes it provides a similar effect as soaking of raw material in a solution of inhibitors at atmospheric pressure. The effectiveness of this method in relation to inhibition of changes in color of fruit and vegetables resulting from enzymatic browning may be connected with the tissue structure, the type of used inhibitors and the conditions of vacuum impregnation.

Literature sources also describe the effect of vacuum impregnation on modification of sensory attributes of the product, such as aroma or taste. An example in this respect is given in a study by Comandini *et al.* (2010) [39]. The authors investigated impregnation of apple slices with green apple aroma. They conducted the impregnation process in vacuum or at atmospheric pressure. Afterwards, the samples were stored in a solution at atmospheric pressure or using ultrasound technology for 2.5, 5.0 and 12.5 min. They found that the best effect is provided by the application of vacuum impregnation and vacuum impregnation combined with ultrasound treatment. Different impregnation behaviors were recorded for alcohols and esters: the content of the former increased even after 5 min of treatment, and the other components increased until 5 min and then decreased, mainly when ultrasound treatment was applied. An advantageous effect of vacuum impregnation on aroma in the case of minimally processed pears was also reported by Perez-Cabrera *et al.* (2011) [35]. When impregnating pear slices with an isotonic solution containing enzymatic browning inhibitors with an addition of calcium, the authors recorded inhibition of the rate of respiration processes in the stored product and thus a lower concentration of anaerobic respiration products (ethanol and acetic aldehyde causing an adverse change in aroma) than in samples impregnated with an isotonic solution or those with no calcium added. In turn, Pino *et al.* (2008) investigated the effect of osmotic dehydration conducted at atmospheric pressure or with the application of vacuum, either continuously or pulsed, on the contents of volatile compounds in guava fruit [64]. The authors carried out the dehydration process at various temperatures (30, 40 and 50 °C) and process duration (1, 2 and 3 h). The greatest losses of volatile compounds were recorded during osmotic dehydration in vacuum applied in both a continuous and pulsed manner at a temperature of 50 °C. The lowest total volatile losses occurred at 30 and 40 °C for up to 3 h under pulsed vacuum or atmospheric pressure.

Changes in product quality are often associated with taste. It seems that this sensory quality attribute may also be modified by vacuum impregnation. It may be applied during the modification of the applied technological operation or as a process preceding freezing, minimal processing or candying. An example is provided by a study by Tamer *et al.* (2013), who applied vacuum impregnation with a solution of sodium chloride or sodium hydroxide and their mixture in order to shorten the length of the debittering process for green table olive cv. “Domat” [40]. As a result of vacuum impregnation, the authors obtained a similar effect to soaking in the solution at atmospheric pressure, while the use of vacuum made it possible to reduce the processing time for the process aiming at the elimination of bitter taste from 15 days to 6 h. In turn, Blanda *et al.* (2008) applied vacuum impregnation to apple slices of cv. Stark Delicious and Granny Smith with a hypertonic aqueous solution containing dextrose, sucrose, ascorbic acid, calcium chloride and sodium chloride [41]. After impregnation the samples were frozen. A significant increase in sweetness was recorded in the apple slices. Samples, which had not been impregnated, were not sensorically acceptable, mostly due to considerable losses of cell sap. During the studies regarding the vacuum impregnation of lychee fruits with a hypertonic solution of sucrose containing cystein, ascorbic acid and 4-hexylresorcinol, Shah and Nath (2008) observed an extension of time, in which taste was considered desirable, by 4 days in comparison to samples impregnated at atmospheric pressure with a solution with no sucrose added [38]. An advantageous effect of vacuum impregnation on sensory attributes, including taste, was also reported by Tapia *et al.* (2003) when vacuum impregnation of melon slices with a sucrose solution (8%, 29% and 50%) was conducted with an addition of calcium ions and zinc [65]. The resulting taste of the product was more acceptable than the taste of fresh raw material. Vacuum impregnation may also modify sensory quality of candied products. A drawback of the candying process is associated with the use of high temperatures during impregnation of raw material with a sucrose solution. This frequently leads to adverse changes in sensory attributes. At lower temperatures this process takes a very long time while providing low efficiency. An example for the application of vacuum impregnation in fruit candying is given in the study by Barat *et al.* (2002) [66]. In their experiments vacuum impregnation of pineapples using a sucrose solution (25° Brix) followed by osmotic dehydration in hypertonic sucrose solutions (55° and 65° Brix) at 15 °C provided a candied product with a better sensory quality, a lower tissue shrinkage and high efficiency of the process in comparison to the process with no vacuum impregnation stage.

It seems that the effect of improving sensory characteristics of fruit and vegetables by vacuum impregnation (especially color and aroma) largely depends on the composition of the impregnating solution. Researchers rarely compare vacuum impregnation with other methods, e.g., soaking or blanching. In many studies the main objective is to determine the effect of the composition of the impregnating solution on the quality of the obtained product. The research of Shah and Nath (2008) and Bland *et al.* (2008) showed that the use of the vacuum impregnation, particularly referring to the color, gives the same result as soaking at atmospheric pressure [38,63]. In this area, there is a need for a broader study, since the effectiveness of vacuum impregnation may vary based on several factors, for example, the differences in porosity and rigidity of the tissue material. The references indicate that using vacuum impregnation for taste enhancement makes it possible to shorten the duration of the technological process [38,40].

## 4. Vacuum Impregnation as a Tool to Modify Health-Promoting Properties of Fruit and Vegetable Products

Similarly to microcapsulation, vacuum impregnation may be employed for as a technique for the application of edible film and coating, in order to produce functional food using physiologically active raw materials. Production of novel functional food with the application of vacuum impregnation facilitates the introduction of e.g., probiotics, vitamins, biologically active compounds and minerals to the fruit and vegetable matrix (Table 2). Literature data mainly concern the kinetics of matrix fortification, potential use of certain active compounds, interactions between tissue and the introduced compound as well as the effect of additives on the rate of respiration processes and potential protection of products against spoilage [4,5,9,67,68].

**Table 2:** Examples of applications of vacuum impregnation to modify health-promoting properties of fruit and vegetable products.

Raw Material	Vacuum Impregnation Solutions Composition	Process Parameters	Effect	References	
apple cylinders	apple juice with an addition of microorganisms *Saccharomyces cerevisiae*, milk with an addition of *Saccharomyces cerevisiae* and *Lactobacillus casei*	*p*_1_ 5 kPa *t*_1_ 10 min *t*_2_ 10 min	over 10^6^ CFU/g *Lactobacillus casei* in air dried (40 °C) product	[4]
pieces of guava and papaya	papaya and guava fruit juices (1—Extracted by blending with water, ratio 1:1; 2, 3—Extracted fruit juices containing 15° and 30° Brix, respectively) with an addition of *Lactobacillus casei* microorganisms	*p*_1_ 5 kPa *t*_1_ 5, 10, 15 min *t*_2_ 10 min	after impregnation: 10^8^ to 10^9^ CFU/g *Lactobacillus casei*, after drying at 40 °C for 36 h: 10^7^ CFU/g *Lactobacillus casei* in impregnated fruits	[69]
apple	isotonic sucrose solution containing 10^8^ CFU/g *Bifidobacterium* ssp.	*p*_1_ 14, 17, 30, 43, 57 kPa	greater incorporation at pressures of 14 and 17 kPa, levels of microorganisms over 10^7^ CFU/g	[70]
apple cylinders cv. Granny Smith	sucrose isotonic solution containing microorganisms *Saccharomyces cerevisiae*, *Lactobacillus acidophilus* and *Phoma glomerata*	*p*_1_ 10, 17, 30, 43, 57 kPa (one vacuum pulse of 2 min)	increase by 0.36 log for *Saccharomyces cerevisiae*, 0.73 log for *Lactobacillus acidophilus* and 1.07 log for *Phoma glomerata* for vacuum impregnated sample in comparison to soaking sample	[71]
apple slices (cv. Fuji)	apple juice diluted with pre-sterilized distilled water (1:1, *v*/*v*, pH 5–5.2) with an addition of *Lactobacillus rhamnosus* (ATCC 7469, in 1:1 (*v*/*v*) glycerol frozen cultures)	*p*_1_ 20 kPa *t*_1_ 15 min *t*_2_ 15 min apple slices were dried by air drying, freeze drying, and a combination of air drying + REV drying	after vacuum impregnation: 10^9^ CFU/gof tissue	[72]
apples cv. Granny Smith (disk-shaped samples)	mandarin juice (pH 5, 8–6, 0) with an addition of *Lactobacillus salivarius* (*Salivarius* spp.)	*p*_1_ 5 kPa *t*_1_ 10 min *t*_2_ 10 min	after vacuum impregnation: 1.51·10^8^ CFU/g *Lactobacillus salivarius* spp. *Salivarius*; the highest microbial content: after 24 h incubation period, pH 6	[73]
apples cv. Granny Smith (disk-shaped samples)	mandarin juice inoculated with *Lactobacillus salivarius* spp. *salivarius* at pH 6 and after 24 h incubation	*p*_1_ 5 kPa *t*_1_ 10 min *t*_2_ 10 min	concentration of microorganisms in the final product: 10^7^ CFU/g	[74]
eggplant fruits and orange peel	isotonic solution of sucrose, iron gluconate and calcium lactate	*p*_1_ 5 kPa *t*_1_ 15 min *t*_2_ 15 min	a mathematical model to determine the concentration of active components in impregnation solution was established in order to formulate functional food with different calcium and iron salts levels	[9]
iceberg lettuce leaves	sucrose aqueous solution of the same *a*_w_ as lettuce leaves used vaccum impregnation reference solution and isotonic solution with an addition of Ca lactogluconate (5.4 g Ca/L of water)	*p*_1_ 50 kPa *t*_1_ 10 min *t*_2_ 10 min	total content of 169 mg Ca per 250 g of impregnated iceberg lettuce leaves	[75]
apple slices cv. Granny Smith	sucrose isotonic solutions with an addition of calcium lactate (44.2 g/L) or ferrous gluconate (1.13 g/L)	*p*_1_ 5 kPa *t*_1_ 10 min *t*_2_ 10 min	after vacuum impregnation: fruits enriched with Ca^2+^ and Fe^2+^ ions respectively	[76]
apple slices cv. Granny Smith	isotonic aqueous solution containing sucrose (*a*_w_ 0.986) and calcium lactate OD in osmotic solution with an addition of 1% calcium salt	*p*_1_ 5 kPa *t*_1_ 10 min *t*_2_ 10 min	an increase in calcium content from 0% to 40% of the recommended daily intake for an adult per 200 g of apples	[77]
fresh-cut apples cv. Fuji	20% diluted high fructose corn syrup (HFCS) or 1% calcium caseinate (CC) aqueous solution with an addition of 0.4% α-tocopherol acetate, 7.5% Gluconal Cal^®^ (GC), and 0.04% zinc lactate (ZL)	*p*_1_ 13.3 kPa *t*_1_ 15 min *t*_2_ 30 min	in 100 g fresh-cut apples the vitamin E content increased more than 100 times, and calcium and zinc contents increased about 20 times compared with unfortified apples	[78]
fresh-cut pears cv. D’Anjou	20% diluted wildflower honey solution with 0.4% to 0.8% α-tocopherol from 3 different sources: α-tocopherol-acetate (VE-acetate), free α-tocopherol (V-OH), or water-soluble α-tocopherol-acetate (VE-H_2_O)	*p*_1_ 10 kPa *t*_1_ 15 min *t*_2_ 30 min	vitamin E content of impregnated pears increased 80 to 100 times and 65% to 80% VE activities were retained during 2 week of storage	[79]
whole potatoes	10% ascorbic acid solution	*p*_1_ 9.33 kPa *t*_1_ 0–60 min *t*_2_ 3 h	after vacuum impregnation the ascorbic acid content of whole potatoes increased ten times (150 mg/100 g fresh weight)	[80]
endive, cauliflower, broccoli, carrots	vacuum impregnation reference solution—aqueous sucrose solutions of the same *a*_w_ as each of the four raw materials; Aloe vera aqueous solution with an addition of 5 and 30 g/L of aloe vera powder (powder dispersed in water), respectively	*p*_1_ 50 kPa *t*_1_ 10 min *t*_2_ 10 min	after vacuum impregnation: incorporation of up to 7 g of Aloe vera in 100 g (dry matter) in broccoli, about 4 g in cauliflower and endive, and about 3 g in carrots	[81]
apples cv. Granny Smith (disk-shaped samples)	mandarin low pulp juice	*p*_1_ 5 kPa *t*_1_ 10 min *t*_2_ 10 min	forty grams of the final product (apple snack) made using mandarin juice provide the same quantity of hesperidin as 250 mL of fresh mandarin juice	[82]
13 apple cultivars (6 mm apple slices)	commercial apple juice (11.1° ± 0.1° Brix) enriched with 0.3% hfv (high in flavonoids) apple peel extract	*p*_1_ 10–80 kPa *t*_1_ 5 min *t*_2_ 10 min	after vacuum impregnation of 13 apple cultivars: quercetin content ranged between 368 and 604 μg/g dry matter	[83]
green apples cv. Orin (apple cubes)	sugar solution (total soluble solids of 50° Brix ) mixed with blackcurrant syrup (47.4°–47.8° Brix). Total soluble solids of the mixture of sugar syrup/blackcurrant syrup of 80%/20%, 70%/30% and 60%/40% were 49.1°, 49.3° and 49.5° Brix, respectively	*p*_1_ 40, 60, 80 kPa *t*_1_ 15, 30, 45 min (following a Box–Behnken response surface methodology design) *t*_2_ 2.5 min	optimized conditions for vacuum impregnation of apple cubes were 18%–20% blackcurrant concentrate level, 77–80 kPa vacuum pressure and 38–45 min vacuum time	[84]
fresh-cut apple cv. Granny Smith (wedges, each *ca.* 10 g)	50% (*v*/*v*) Mexican or 50% (*v*/*v*) Argentinean honeys, distilled water (control sample)	*p*_1_ 70 kPa *t*_1_ 10 min *t*_2_ 10 min	less acceptable in terms of sensory qualities than their fresh-cut counterparts, total polyphenol content and antioxidant activity values in vacuum impregnated products were lower than in fresh-cut samples	[85]

*p*_1_—vacuum pressure in the VI process; *t*_1_—time in reduced pressure; *t*_2_—time in atmospheric pressure.

### 4.1. Probiotics Introduced to the Fruit and Vegetable Matrix

Probiotics are selected strains of living bacteria, which exhibit an advantageous effect on human health after consumption. Probiotic bacteria have been used in food to provide health benefits for approx. 20 years. Well-known probiotics include lactic acid bacteria, belonging to the genera *Bifidobacterium* and *Lactobacillus*. Metabolites of probiotic bacteria stimulate the immune system, promote peristalsis and intestinal secretion, while also exhibiting antibacterial and antiviral action, thus inhibiting the development of pathogenic bacteria and reducing the amount of produced toxins [86]. Attempts to apply vacuum impregnation in order to introduce probiotics to the fruit and vegetable matrix were associated with the extension of the range of probiotic foodstuffs. Most products with an addition of probiotics are dairy products, having two basic disadvantages related with their consumption, *i.e.*, lactose intolerance and high cholesterol content. Moreover, consumption of probiotic products is limited in view of the increasing numbers of vegetarians. This is the direct reason for the search for new possibilities to add probiotics, e.g., to raw materials, such as fruit and vegetables [87,88]. Probiotic food most typically contains microorganisms from the genera *Lactobacillus, Bifidobacterium* and *Saccharomyces* [67,86,89]. Betoret *et al.* (2003) applied vacuum impregnation of apples with apple juice supplemented with *Saccharomyces cerevisiae* or milk inoculated with *Saccharomyces cerevisiae* and *Lactobacillus casei* [4]. Impregnation facilitated the effective introduction of probiotics to apple tissue, providing the content of microorganisms in the product after convection drying (air drying) at 10^6^–10^7^ CFU/g. This is equivalent to the level of bacteria in dairy products. Similarly, Krasaekoopt and Suthanwong (2008) obtained the level of microorganisms in fruit after air drying at 10^7^ CFU/g during the vacuum impregnation of guava and papaya fruits using *L. casei*, which makes this product probiotic food [69]. Maguina *et al.* (2002) impregnated apple slices with a sugar solution containing approximately 10^8^ CFU/g of *Bifidobacterium* ssp. [70]. The authors applied vacuum fortification of tissue at different pressure levels, in each case obtaining the level of bacteria over 10^7^ CFU/g. Rodriguez (1998) applied vacuum impregnation with isotonic sugar solutions supplemented with *Saccharomyces cerevisiae*, *Lactobacillus acidophilus* and *Phoma glomerata* [71]. The matrix consisted of cylindrical pieces of Granny Smith apples. The author applied one vacuum pulse at five levels of pressure and soaking in the same solutions with no vacuum application. In the case of vacuum impregnation, the author observed a microbial growth rate higher by 0.36 log for *S. cerevisiae*, 0.73 log for *L. acidophilus* and 1.07 in the case of *P. glomerata* in comparison to soaked samples. A significant effect of the content of extract in the impregnating solution on the level of microorganisms introduced to the tissue was found in most of the presented studies. The best results were obtained during the application of isotonic or almost isotonic solutions. The use of hypo- and hypertonic solutions resulted in significantly lower levels of microorganisms in the final product.

Most of the conducted studies present vacuum impregnation as an effective tool in the production of probiotic food based on the matrix of fruit and vegetable materials, as an alternative to dairy products. At the same time, a significant aspect is also associated with the method of fruit and vegetable tissue preservation after the introduction of microorganisms, facilitating the maintenance of their high levels in the final product. The most frequently used method in this respect, with potentially the greatest applicability, is drying, particularly air drying and freeze-drying. Noorbakhsh *et al.* (2013) introduced bacteria *Lactobacillus rhamnosus* to the tissue of apple slices together with apple juice diluted with water at a 1:1 ratio (pH 5–5.2) [72]. Vacuum impregnated apple slices were air dried, freeze-dried and dried in a process combining air drying and radiant energy vacuum drying. Initially, the *L. rhamnosus* population in apple slices tissue after impregnation was at 10^9^ CFU/g. The freeze-drying process was most effective in protecting bacteria in comparison to the other two drying methods, reducing the microbial population by 1.1 log. In turn, a combination of air drying and radiant energy vacuum drying resulted in a smaller reduction of the level of microorganisms during room temperature storage in enriched apple snacks. Additionally, in a study by Betoret *et al.* (2009), a combination of vacuum impregnation and drying provided a probiotic fruit product containing microorganisms at a level comparable to that in probiotic dairy products [73]. Those authors applied a combination of vacuum impregnation of apple slices with mandarin juice supplemented with *Lactobacillus salivarius* (spp. *salivarius*) with their convection drying at high temperature (hot air drying techniques), proposing this product as snacks and an addition to breakfast cereals. At the same time, the introduced probiotics were investigated as potential factors inhibiting the development of *Helicobacter pyroli*. In a continuation of this research Betoret *et al.* (2012) used the produced apple snacks, containing 10^7^ CFU/g dry matter of bacteria *L. sallivarius* ssp. s*alivarius* introduced with mandarin juice, in clinical trials conducted *in vivo* [74]. In a group of five dyspeptic children given apple snacks the authors recorded results suggesting a positive effect suppressing *H. pyroli*, measured based on standard infection indexes.

The introduction of prebiotics to apple tissue seems to be an interesting idea. As carbohydrates naturally found in food, they may promote the growth and activity of introduced probiotic microorganisms [86]. Matusek *et al.* (2008) introduced fructo-oligosaccharides in a 60% solution of a commercial preparation (Beneo™ P95, Beneo GmbH, Mannheim, Germany) produced by hydrolysis of inulin from chicory to the tissue of apples (Idared) [90]. The authors used vacuum impregnation on apple cubes previously blanched at a temperature of 70 °C for 5 min. The impregnation process was run at 75 kPa for 5 min, followed by osmotic dehydration. Those authors recorded no differences in the rate of diffusion of fructo-oligosaccharides to tissue after the application of vacuum impregnation in comparison to samples osmotically dehydrated in a solution with no vacuum applied.

### 4.2. Enrichment with Minerals and Vitamins

Minerals are essential for the appropriate functioning of the human organism. Their deficiency maintained over a longer period may be involved in the etiology of certain metabolic civilization-related diseases. The effect of inadequate intake of minerals on the development of cardiovascular diseases, osteoporosis, diabetes or certain cancers has been well-documented [91,92,93,94,95]. Most fruit and vegetables are characterized by low contents of minerals, e.g., calcium, zinc and iron. Enrichment of fruit and vegetables with minerals, frequently including calcium, has been recently considered as a process to produce food with an enhanced nutritive and health-promoting value. Calcium, as an integral component of the organism, influences numerous exo- and intracellular processes, e.g., muscle contractibility, nerve conduction, secretion and action of hormones, as well as blood clotting. Calcium deficiency is frequently associated with epidemiology of several chronic diseases, including osteoporosis, hypertension and colon cancer [96]. Calcium uptake from the diet, similarly as in the case of probiotic microorganisms, is mostly connected with dairy products, which—as it has been indicated above—may have numerous adverse aspects. Fito *et al.* (2001) were the first to test the introduction of minerals to the fruit and vegetable matrix using vacuum impregnation [8]. Those authors created a mathematical model determining the level of minerals in the impregnating solution, which would provide a product covering 20%–25% dietary reference intake as a result of enrichment of 200 g sample as a food ration. The mineral concentration in a vacuum impregnated product is calculated on the basis of ion concentration, density of the initial sample and the impregnation solution. The solubility of different mineral salts determines the maximum value of mineral concentration and the possibilities of mineral concentration to increase for a specific porosity in the product. This is especially relevant for calcium, due to the high value of the recommended daily intake (RDI). The factors affecting the level of minerals should also include parameters directly related to the efficiency of vacuum impregnation: effective porosities of fruit and vegetable tissue as well as volumetric deformation coefficient. Based on the parameters mentioned above, also it is possible to calculate the mass of the sample containing the recommended daily intake of minerals [8]. Fito *et al.* (2001) applied vacuum impregnation to introduce calcium and iron ions to the tissue of sliced eggplant and orange rind, similarly as it was in a study by Barrera *et al.* (2004) for apple tissue [8,76]. Introduction of Ca^2+^ and Fe^2+^ to apple tissue was connected with osmotic dehydration, however, low concentrations of introduced ions did not change the kinetics of the process and the effectiveness of calcium and iron introduction by vacuum impregnation was comparable in hypo- and hypertonic solutions [76]. Gras *et al.* (2011) enriched iceberg lettuce with calcium using vacuum impregnation and obtained calcium content similar to that in dairy products at 160 mg Ca^2+^ per 250 g of lettuce, making the product an alternative source of calcium [75]. Barrera *et al.* (2009) applied osmotic dehydration to calcium enriched slices of apple cv. Granny Smith [77]. Vacuum impregnation alone made it possible to increase the content of calcium from 0% to 40% of the recommended daily uptake for adults in 200 g of apple. Calcium ions were introduced to an isotonic sucrose solution in the form of calcium lactate, in an amount calculated based on an equation proposed by Fito *et al.* (2001), after which apples were osmotically dehydrated in a sucrose solution (extract 50° Brix) containing an addition of calcium [8]. A decrease in water content during osmotic dehydration resulted in a partial loss of calcium ions; however, their level upon the completion of the process was high enough to establish the product as enriched. Moreover, osmotic dehydration turned out to be a good tool in the extension of shelf life of enriched fruit.

Apart from minerals, vitamins may also be introduced to the fruit and vegetable matrix. According to Leonard *et al.* (2004), the bioavailability of vitamin E is significantly greater when applied in the form of food rich in this vitamin than in the form of capsule supplements [97]. In this respect, the introduction of vitamin E to fruit and vegetables, particularly as they typically contain its slight amounts, seems to be an interesting alternative to supplements. During the enrichment of fresh-cut apples with vitamin E as well as calcium and zinc Park *et al.* (2005) obtained a 100-fold greater content of vitamin E per 100 g of apples and approximately 20-fold higher contents of calcium and zinc in apples in comparison to apples before impregnation [78]. For this purpose the authors applied vacuum impregnation in a 20% corn syrup solution. Lin *et al.* (2006) introduced vitamin E together with a 20% solution of polyfloral honey to the tissue of pears [79]. The process of vacuum impregnation provided an 80–100-fold increase in vitamin E content in pears. Moreover, 65% to 80% vitamin E were preserved over the course of 2-week storage of fruit at a temperature of 2 °C. A study by Han *et al.* (2004) also confirmed the stability of vitamin E during storage of fruit enriched using vacuum impregnation [98]. Moreover, the authors suggested that α-tocopherol acetate is a highly stable form of vitamin E, resistant to light, air and heat. Contents of vitamin E in enriched fresh and frozen strawberries did not change significantly during their two-week storage at 2 °C and 6-month frozen storage [98]. The study by Bruno *et al.* (2005) implies that vitamin E (α-tocopherol) introduced to apples may be absorbed by the human organism, while a simultaneous supply of a slight amount of fat with the diet enhances absorption of α-tocopherol [99]. Hironaka *et al.* (2011) investigated the possibility to enrich whole potato tubers with ascorbic acid [80]. For this purpose the authors applied vacuum impregnation with a 10% ascorbic acid solution. The authors recorded an increase in the content of ascorbic acid following vacuum impregnation to a max. content of 150 mg/100 g fresh weight. Prolongation of the impregnation time from 15 to 60 min at the pressure of 9.3 kPa resulted in a more than three-times increase of the content of ascorbic acid in the impregnated sample. A 25-min steaming caused a decrease in the content of ascorbic acid in potatoes. However, potatoes prepared such way may provide a 90%–100% recommended daily allowance for adults (100 mg). Moreover, the content of ascorbic acid after 14 days of storage in vacuum impregnated raw potato tubers at 4 °C was at 50 mg/100 g fresh weight Based on available biochemical, clinical and epidemiological tests, the recommended daily intake (RDI) for vitamin C is currently set at 80–100 mg/day for adults. It is the level facilitating cell impregnation and a reduced risk of cardiac disease, cerebral stroke and cancer in healthy individuals. The metabolic role of ascorbic acid connected with reactions of oxidation and reduction, in which ascorbic acid is reversibly oxidized to dehydroascorbic acid [100,101].

In order to introduce the mineral, most authors applied a vacuum pressure of 5–50 kPa for 10–60 min, whereas the pressure value was related to the type of raw material and, in many cases, its effect on the process efficiency has not been studied. According to Alzamora *et al.* (2004) the kinetics of incorporating Ca^2+^ to an apple matrix was dependent on the vacuum pressure during impregnation and the structure of the fruit tissue [67]. On the basis of Fito *et al.* (2006) and Mújica Paz *et al.* (2003) it can be concluded that the increase in vacuum pressure contributes to the introduction of a higher concentration of the compound into the tissue, where it is largely dependent on raw material [6,45].

### 4.3. Other Applications of Vacuum Impregnation to Enrich the Fruit and Vegetable Matrix

Several studies using vacuum impregnation as a tool to produce functional food concerned the introduction of different enriching compounds coming from natural raw materials and products (e.g., mandarin juice, aloe powder, red currant concentrate and honey) to the food tissue [79,81,82,83,84,85]. Most frequently, the aim of such studies was to determine the potential enrichment using vacuum impregnation, select a technique for product preservation, as well as to determine its effect on contents of introduced compounds.

Sanzana *et al.* (2011) tested the potential for the production of functional food by vacuum impregnation of broccoli, cauliflower, endive and carrot with a solution supplemented with aloe powder (Aloe barbadensis, Terry laboratories, Malbourne, Australia) at 30 g/L [81]. The authors introduced considerable amounts of aloe (7 g/100 g dry matter to broccoli, 4 g/100 g dry matter to cauliflower and endive, 3 g/100 g dry matter to carrot), while the introduced amount was dependent on the porosity of tissue characteristic of individual raw materials. Aloe vera is used in treatment of many diseases, including dermatoses. It was shown that aloe gel obtained from leaf flesh reduces blood glucose level in diabetics, lowers lipid level in patients with hyperlipidemia, while it also inhibits secretion of pepsin and hydrochloric acid, thus preventing the development of chronic gastric ulcer disease [102]. In a study by Sanzana *et al.* (2011), apart from the introduction of aloe in the production of functional food, the effect of applied impregnation on the rate of respiration processes was determined in raw materials, which may be a factor extending shelf life of enriched vegetables [81]. At the same time, vacuum impregnation with a solution supplemented with aloe had a varied effect on the respiration processes and respiratory quotient of tested vegetables, making it difficult to draw generalized conclusions. Betoret *et al.* (2012) applied vacuum impregnation to introduce homogenized mandarin juice with a low fruit flesh content to apple snacks [82]. The primary aim of the studies was to investigate the possibility to apply the product enriched with bioactive compounds found in mandarin juice, mainly flavonoids. The authors obtained the content of hesperidin in 40 g of the enriched product equivalent to that in 250 mL of mandarin juice. The study used the same vacuum impregnating parameters for all samples (Table 2), however, significant differences in the content of flavonoids were noted in samples impregnated with tangerine juice that were subjected to homogenization at different pressures (10,000–25,000 kPa). On the other hand, higher pressure homogenization of the juice resulted in an increase of narirutin only, in the content of impregnated apple. The current state of knowledge on the biological activity of flavonoid compounds clearly indicates that their positive effect on the human organism results mainly from antioxidant properties. The capacity of flavonoids to scavenge reactive oxygen species (ROS) and chelate transition metals may have a significant role in pathological conditions (e.g., inflammations, atherosclerosis, diabetes, neurodegenerative diseases or cancer), which are accompanied by oxidative stress [103,104]. In a continuation of studies by Betoret *et al.* (2012) apple snacks vacuum impregnated with mandarin juice were administered to obese children in order to alleviate inflammatory conditions and improve the antioxidant capacity of the organism [82,105]. A considerable improvement was observed in systolic blood pressure and the lipid profile following the treatment period. The authors concluded that the addition of the product to diet contributed to an alleviation of oxidative stress and the inflammatory condition, as well as several other risk factors connected with atherosclerosis in the examined obese children.

Plant flavonoids (mainly quercetin and rutin) additionally exhibit a protective action towards vitamins C and E. Their capacity to chelate ions of copper and other transition metals inhibits e.g., oxidation of ascorbate [106]. Quercetin glycosides were introduced to apple tissue by Schulze *et al.* (2012) [83]. For this purpose, the authors applied vacuum impregnation with apple juice with an addition of cider apple skin extract (Val de Vire Bioactives, Condé sur Vire, France). Impregnation of 13 apple varieties provided quercetin content of 368–604 μg/g d.m., while the content was correlated with the hardness of native apples. The authors obtained a higher level of quercetin during the impregnation with a solution of a low extract content (11.1° Brix) in comparison to solutions with higher viscosity containing an addition of apple pectin. Schulze *et al.* (2014) also enriched apple slices with quercetin derivatives before their freeze-drying, microwave-vacuum drying and convection drying [107]. Quercetin and its derivatives were introduced similarly as in the previous studies with apple juice containing an addition of apple skin extract. The authors did not record losses of introduced polyphenolic compounds during freeze-drying or microwave-vacuum drying, while convection drying caused losses of quercetin and its derivatives amounting to 44%.

Diamante *et al.* (2014) introduced a black currant extract to apple cubes by vacuum impregnation [84]. This procedure may cause an increase in the content of polyphenolic compounds in apple tissue, mainly anthocyanins, thus modifying fruit color as well as vitamin C content, since black currant is a good source of this compound. The authors investigated the effect of extract concentration, the level of pressure and the duration of its application on both mass transfer and nutritive value of apple cubes by applying the response surface method. In the process of vacuum impregnation they used high fructose corn syrup with extract content of 50° Brix and a black currant concentrate mixed in the following proportions [%]: 90/10, 85/15, 80/20 and pressure within the range of 40–80 kPa in time ranging from 15 to 45 min. Higher pressure and longer duration resulted in a greater uptake of extract compounds, but not a high black currant content. A higher antioxidant activity was obtained using medium and high concentrations of black currant concentrate, while the highest content of vitamin C was recorded at a medium content of black currant in the impregnating solution. Optimal parameters of vacuum impregnation were specified based on the analyses, promoting the introduction of such amounts of black currant concentrate, which significantly enhanced the nutritive value of produced apple cubes.

Roβle *et al.* (2011) applied vacuum impregnation of fresh-cut apple wedges in order to enrich them with honey, additionally applying an addition of browning inhibitors and subjecting the enriched fruit to osmotic dehydration [85]. The authors evaluated the effect of impregnation with honey on the physico-chemical properties, sensory attributes and antioxidant capacity of apples stored for 7 days at a temperature of 2–4 °C. Honey is used in the prevention of many diseases and increasingly often plays a special role in human nutrition, particularly thanks to the content of polyphenolic compounds and choline. However, in this case the introduction of honey to apple tissue caused both a reduction in sensory quality in comparison to apples not subjected to treatment, and lower contents of polyphenolic compounds and antioxidant activity. Losses of phenolic compounds and a reduction of antioxidant activity may result from osmotic dehydration of tissue, which suggests a need to apply another method of product preservation, e.g., drying, as in the case of other enriched fruit and vegetables.

## 5. Conclusions

Currently, the food processing sector is searching for new methods to modify the functional properties of food. Plant tissue is a perfect matrix, which could be a carrier of bioactive compounds and nutrients. Simultaneously, it is important to ensure that such foods exhibit preferred sensory attributes and storage stability. The use of the vacuum impregnation technique allows for the introduction of different ingredients to the raw material originating from plants, which could influence their stability, taste, texture and nutritional value.

The use of vacuum impregnation treatment may decrease the pH value of food, which facilitates its later fixation and increases storage time. Acidification with the use of this technique is characterized by shorter process duration and higher efficiency compared to e.g., soaking or blanching. The combination of vacuum impregnation with osmotic dehydration, often called pulsed vacuum osmotic dehydration (PVOD), allows for an enhancement of mass transfer and induces less structural changes, especially in the first stage of the process. Removal of gas from the tissue during soaking and simultaneous introduction of a solution contributes to an inhibition of browning processes, improves the heat transfer in the material and has a positive influence on the structure during latter freezing of the product. The vacuum impregnation technique may also be used in order to modify plant products into a source of components, which were not previously found in plants or are present at marginal levels under natural conditions (e.g., calcium and iron ions or probiotics). Such products may be valuable diet enrichment for particular groups of consumers, e.g., vegetarians or lactose intolerant people. Additionally, no loss of bioactive compounds already present in the fruit and vegetables was observed after the use of vacuum impregnation (e.g., there is no leaching, which commonly occurs during osmotic dehydration). The vacuum impregnation technique does not require the use of hypertonic solutions, since the process is efficient even at low concentrations of the compound used for the impregnation. Additionally, the duration is notably reduced and the process may be conducted at low temperatures.

The process conditions and the composition of the solution should be optimized depending on the type of raw material and the expected effect. Generally, the use of lower pressure and prolonged process time contribute to an increased rate and intensity of impregnation. However it should be noted, that the soft tissue of some fruit may become damaged and thus inhibit the introduction of the solution into the matrix. Modification of properties or introduction of novel components into the material due to the use of vacuum impregnation may be advantageous during the latter processing steps or improve the quality of the end product.
